# Biochemical and Biophysical Characterization of the Caveolin-2 Interaction with Membranes and Analysis of the Protein Structural Alteration by the Presence of Cholesterol

**DOI:** 10.3390/ijms232315203

**Published:** 2022-12-02

**Authors:** Berta Gorospe, José J. G. Moura, Carlos Gutierrez-Merino, Alejandro K. Samhan-Arias

**Affiliations:** 1 LAQV-REQUIMTE, Departamento de Química, Faculdade de Ciências e Tecnologia, Universidade Nova de Lisboa, 2829-516 Lisbon, Portugal; 2 Research Institute of Molecular Pathology Biomarkers, University of Extremadura, 06006 Badajoz, Spain; 3 Departamento de Bioquímica, Universidad Autónoma de Madrid (UAM), C/Arturo Duperier 4, 28029 Madrid, Spain; 4 Instituto de Investigaciones Biomédicas ‘Alberto Sols’ (CSIC-UAM), C/Arturo Duperier 4, 28029 Madrid, Spain

**Keywords:** caveolin-2, caveolin-1, cholesterol, membrane interaction, secondary structure prediction

## Abstract

Caveolin-2 is a protein suitable for the study of interactions of caveolins with other proteins and lipids present in caveolar lipid rafts. Caveolin-2 has a lower tendency to associate with high molecular weight oligomers than caveolin-1, facilitating the study of its structural modulation upon association with other proteins or lipids. In this paper, we have successfully expressed and purified recombinant human caveolin-2 using *E. coli*. The structural changes of caveolin-2 upon interaction with a lipid bilayer of liposomes were characterized using bioinformatic prediction models, circular dichroism, differential scanning calorimetry, and fluorescence techniques. Our data support that caveolin-2 binds and alters cholesterol-rich domains in the membranes through a CARC domain, a type of cholesterol-interacting domain in its sequence. The far UV-CD spectra support that the purified protein keeps its folding properties but undergoes a change in its secondary structure in the presence of lipids that correlates with the acquisition of a more stable conformation, as shown by differential scanning calorimetry experiments. Fluorescence experiments using egg yolk lecithin large unilamellar vesicles loaded with 1,6-diphenylhexatriene confirmed that caveolin-2 adsorbs to the membrane but only penetrates the core of the phospholipid bilayer if vesicles are supplemented with 30% of cholesterol. Our study sheds light on the caveolin-2 interaction with lipids. In addition, we propose that purified recombinant caveolin-2 can provide a new tool to study protein–lipid interactions within caveolae.

## 1. Introduction

Caveolae was one of the first plasma membrane micro-domains described in epithelial cells by George Palade in 1953 [1]. They were early defined as wide pits presented at the cellular plasma membrane representing 30–70% of the total plasma membrane surface in some cases, as accounts in vascular endothelial cells and adipocytes [2]. Caveolae are enriched in protein and lipids, such as sphingolipids and cholesterol, whose presence is key for some of the caveolae’s signal transduction functions, including mechano-protection, mechano-sensing, endocytosis, oncogenesis, and the uptake of viruses and pathogenic bacteria [2].

Caveolin (Cav) is the major protein forming the caveolae [3]. Although Cav participates in membrane invagination formation [3], other functionalities have been reported for these proteins [4,5]. In order to understand the differential functionality of Cav isoforms in the interaction with other proteins or lipids partners, structural studies are demanded. In this sense, some of the structural features of Cav domains have been defined during the last decades using Cav-1 as a model, a predominant isoform in all tissues that can induce caveolar biogenesis [6]. In the case of Cav-2, evidence suggests signaling properties in the regulation of cells and tissues in a tissue type-specific manner, in some cases by showing cellular properties more emphasized than those found for Cav-1 [7]. In addition, for the case of the third Cav isoform named Cav-3, a tissue expression specificity associated with the muscle has been found [6]. In homology, Cav-1 and -3 show a 65% identity and 85% similarity in their protein sequence [8], and human Cav-2 shows 38% identity and 58% similarity with human Cav-1 [9]. The high homology between Cav-1, Cav-2, and Cav-3 sequences guided the research focused on the characterization of Cav-1 structure as the most characteristic and specific signature of lipid rafts rather than performing depth structural studies of the other isoforms. Cav-1 oligomerization occurs in the oligomerization domain (OD) (residues 61–101) [10,11], where the scaffolding domain (CSD) of the protein (residues 82–101) is also present [12]. These domains partially overlap with the transmembrane domain (TM) (96–131) [13]. In addition, an intramembrane domain (IMD) formed by residues 102–134 is proposed as responsible for an uncompleted hairpin loop inserted in the phospholipid bilayer [14] that also overlaps with the TM. Cholesterol-binding to the Cav-1 is ascribed to a cholesterol-binding domain (CRAC) [15,16] located in the TM region. Regarding cholesterol-binding to some proteins, another cholesterol-binding domain has been ascribed to a different motif besides CRAC [16,17]. An alternate cholesterol domain also represents the CARC sequence that exhibits the opposite orientation along the polypeptide chain [17]. Only a few of these described Cav-1 domains have been identified for the rest of the Cav isoforms [18,19,20,21,22,23]. In terms of function in some cases, some Cav isoforms work synergistically [24], although a differential regulatory role has been found for the same domains of Cav-1, -2, and -3 in some situations [12,25]. Using peptides derived from Cav-2 and -3 that correspond to the 82–101 CDS domain of Cav-1, inhibition of the c-Src tyrosine kinase auto-activation has been described for Cav-1 and -3 but not found for Cav-2 derived peptides [12]. Additionally, despite the sequence similarities between Cav-1 and -2, they can differentially modulate GTPases activity [7,9]. Some peptides derived from Cav-2 show a GTPase-activating protein-like activity that behaves differently than those associated with the same sections of Cav-1 [9]. Indeed, the functional interaction of Cav-1 with purified trimeric G-proteins suppressing basal GTPase activity has been reported [26]. 

A role in ROS production and apoptosis signaling has been associated with the neuronal caveolae, where cytochrome b_5_ reductase (Cb_5_R) can produce superoxide anion in the absence or presence of protein partners [27,28,29]. A possible role of the proteins located in these domains, including Cav-1 and -2, has been suggested in ROS regulation and cell death [27,28,30,31]. Indeed, the inhibition of the synaptic plasma membrane vesicles NADH oxidase activity associated with Cb_5_R with antibodies against Cav-1 and -2 suggests a possible control of this activity by Cav isoforms [30]. Cav-1 has been identified as the most enriched plasma membrane Cav isoform in lipid rafts [8]. The regulation of Cb_5_R activity by other Cav isoforms enriched in other locations has not been discarded. One of these examples is Cav-2, which has been mainly confined at the Golgi reticulum but can redistribute into the plasma membrane rafts when co-expressed with Cav-1 [32]. Localization at this organelle has been attributed to the Cav-2 scaffolding domain [19].

Moreover, Cav-2, unlike Cav-1, can also target lipid droplets, as shown in overexpression experiments [33]. This behavior has been attributed to inefficient protein transport through the secretory pathway [32]. In addition, some Cav isoforms can be the transcriptional target for some regulatory factors [34]. A three to five-fold increase in Cav-2 expression was induced by activation of the peroxisome proliferator-activated receptor-γ (PPARγ) upon a drug-induced differentiation therapy in carcinoma cell lines [34]. Other studies have shown that Cav-2 phosphorylation at Y19 is critical for its interaction with phospho-ERK in human insulin receptor-overexpressed rat 1 fibroblast (Hirc-B) cells [35]. In this study, a re-localization of the complex towards the nucleus is controlled by insulin. Further research showed that the Cav-2 domain S144-V155-S156 domain on the C-terminal region is a nuclear trafficking signal in Cav-2 and key in the insulin-induced phosphorylation and nuclear targeting of ERK and Cav-2 [18].

Electron microscopy studies showed that intracellular vesicle formation was -specific with the Cav isoform present in the membrane [36]. A failure to achieve vesicle formation in the presence of Cav-2 allowed some researchers to propose an accessory role of this protein concerning Cav-1 and to suggest a dependence upon Cav-1 for Cav-2 to localize in the cholesterol/sphingolipid-rich micro-domains using NIH 3T3 cell lines harboring Cav-1 [36]. This study correlates with other studies indicating that Cav-2 mainly exists as a monomer or dimer [9,37] and the requirement for Cav-1 to assemble in high molecular weight complexes [37], which contrasts with the Cav-1 ability to form homo-oligomers of ≈350 kDa by itself [36,38,39]. Although Cav-2 overexpressed in cells cannot form intracellular vesicles as Cav-1 does, the similarity between Cav-1 and -2 sequences allows hypothesizing that Cav-2 should directly interact with cellular lipid membranes.

On these grounds, the major aim of this work is to study the putative interaction of Cav-2 with lipid bilayers of egg lecithin with different content on cholesterol and the effects of this interaction on Cav-2 structure to experimentally assess the possible use of this Cav isoform as a model system to study protein–lipid interactions within the caveolae. 

## 2. Results

### 2.1. Purification of Human Recombinant Cav-2

**Figure 1:**
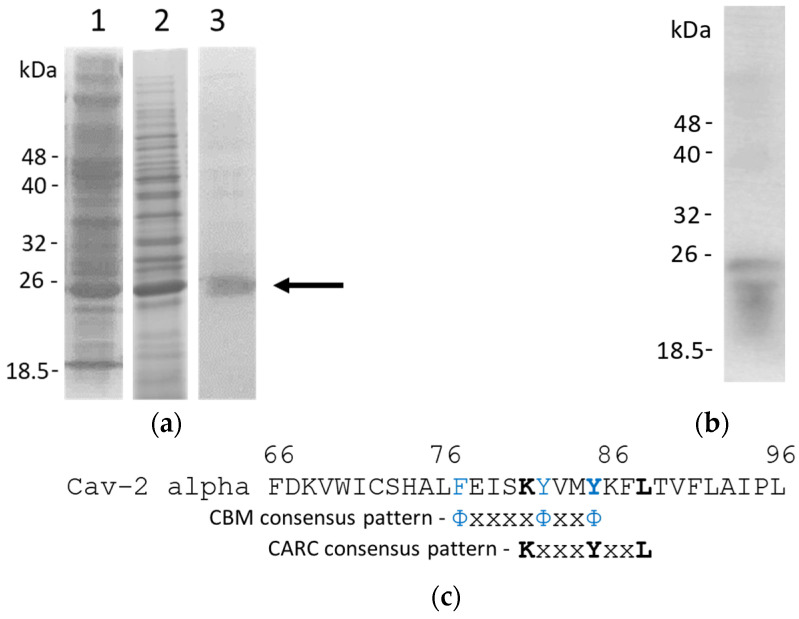
Purification and some structural features of Cav-2. SDS-PAGE electrophoresis of different samples obtained during the purification procedure (panel **a**): wholes lysate (lane 1), Superdex G75 (lane 2), and Nickel Sepharose (lane 3). Samples were loaded onto a 12.5% polyacrylamide gel and run at a constant voltage of 90 V. Gels were sustained to the staining/distaining procedure with Coomassie blue. The arrow indicates the band that corresponds to Cav-2. A lysate prepared in these conditions was subjected to Western blotting with the procedure indicated in the Materials and Methods section to prove the protein expression in bacterial cultures after induction with IPTG. The result of the Western blotting is shown in (panel **b**). The identified CARC and caveolin-binding motifs (CBM) of CAV-2. CARC and CBM motifs were identified during the sequence analysis and comparison with CAV-1 and other proteins containing these domains, as described in the text (pane **c**). Aromatic residues are indicated in blue as part of the CBM motif, and the critical residues of the Cav-2 CARC domains are labeled in bold.

The alpha isoform of human recombinant Cav-2 has been overexpressed in E. Coli and purified as described in the Materials and Methods. SDS-PAGE was used to track the purification process as shown in Figure 1a, where samples from the initial whole lysate and those obtained after chromatography purification using Sephadex G75 and Nickel Sepharose (lanes 1, 2, and 3, respectively) were loaded onto the gel and run. As observed in this figure, the more enriched band in all samples (indicated by an arrow) corresponds to Cav-2. We calculated an average molecular weight close to 24 kDa based on relative mobilities regarding molecular markers. This band gets enriched during the purification process. We obtained the pure protein with some minor higher molecular weight fuzzy contaminants after the Nickel Sepharose chromatography (Figure 1). Table 1 summarizes the protein yield achieved in each purification step.

To further confirm that Cav-2 was overexpressed in bacteria, a sample from the whole lysate was subjected to a Western blot using a goat anti-Cav-2 antibody (Santa Cruz, CA, USA, #sc-1858), as described in the Materials and Methods section. Figure 1b shows Western blotting with the whole bacterial lysates revealed with a primary anti-Cav-2 antibody and a secondary anti-goat HRP antibody. As indicated, the anti-Cav-2 antibody can recognize Cav-2 overexpressed in the whole lysates. 

### 2.2. Structural Predictions Based on the Cav-2 Amino Acid Sequence

#### 2.2.1. Homology Analysis

The sequence of the alpha Cav-2 isoform is shown in Supplementary Figure S1 and this sequence was used to obtain structural information by comparing this sequence with other Cav isoforms. The sequence of Cav-1 human isoforms (α- and β- isoforms; Unitprot #Q031352 and # Q03135-1, respectively) and Cav-2 human isoforms (α- and β- isoforms; Unitprot# P51636-1 and t# P51636-2, respectively) and Cav-3 (Unitprot# P51636-3)] were aligned as indicated in the Materials and Methods section (Supplementary Figure S2), by uploading the sequence to the Mutalin interface alignment server. 

The areas of high consensus (>90% of homology) are shown in red, and the low consensus sequences (<50% homology) are shown in blue (Supplementary Figure S2).

The comparison between the Cav-1 and Cav-2 isoforms sequence showed a high homology between isoforms, especially in the residues 15–63 of the amino-terminal region. The main difference between these sequences is the lack of the first 23 amino acids, in the case of the Cav-1 β isoform in respect to the other sequences, and the lack of the last 40 amino acids of the carboxylic terminal end of the Cav-2 isoform C, in comparison to the rest of the sequences. Regarding the Cav-2 alpha isoform studied in this work, we observed the presence of a CBM motif (ΦxxxxΦxxΦ, where Φ is an aromatic and x an unspecified amino acid)) associated with the amino acids F77EISKYVMY85 (Figure 1c). Moreover, although some similarities have been ascribed to a domain in the Cav-2 sequence (I79-F87) with the Cav-1 CRAC domain [40], we identified that this sequence is part of a CARC domain [16,17] when the leucine 88 is incorporated in the reading sequence. The residues forming the identified CARC domain are K81YVMYKFL88. Some of the residues forming part of these domains are also part of the CBM motif of Cav-2 (Figure 1c).

#### 2.2.2. Prediction of Intrinsically Disordered Segments

**Figure 2:**
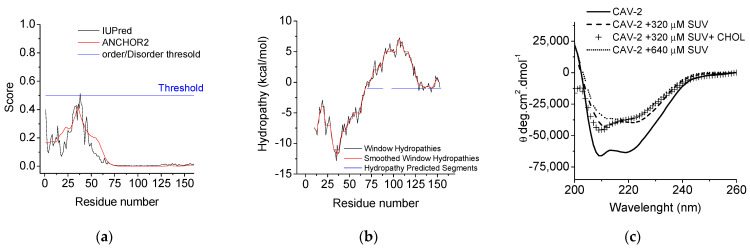
Prediction of intrinsically disordered and hydropathy segments and measurement of Cav-2 far-UV CD spectra in the presence of SUVs. Prediction of intrinsic disorder (panel **a**) and hydropathy segments (panel **b**) were calculated as indicated in the Materials and Methods using the sequence of alpha isoform of Cav-2. Far-UV CD spectra of Cav-2 measured in the presence of SUVs with different lipid compositions and concentrations are shown in (panel **c**). Measurements were carried out at 25 °C with Cav-2 (32 μM) in sodium phosphate buffer 25 mM at pH 7.0 (continuous line) or in the presence of SUVs (320 μM), SUVs (320 μM) + cholesterol 5% and SUVs (640 μM) (crosses, dashed and dotted line, respectively). CD spectra were recorded 205 to 260 nm (far-UV), using a quartz cell of 0.1 mm, respectively. The DSC spectra shown in this figure are representative of triplicate experiments.

IUPred2 and ANCHOR2 web-based implementation predictors [41] were used to identify the intrinsically disordered protein regions of Cav-2 (Figure 2a). The disorder degree was shown on a normalized scale from 0 (ordered) to 1 (disordered). This analysis identifies the protein regions that do or do not adopt a stable structure depending on the redox state of their environment [41]. ANCHOR2 supersedes its previous version and predicts protein binding regions that are disordered in isolation but can undergo disorder-to-order transition upon binding interaction with partners [42]. We obtained very similar results with both servers with a higher degree of disorder in the Cav-2 amino terminal end from the amino acid region from 1 to 74, with the higher disorder score value close to a 0.5 for the amino acid region from residues 30 and 50 (Figure 2a). This result correlates with the presence of 46% of disordered protein. Our data indicate that the most disordered region of the protein was found in the amino acid region from residues 25 to 50. None of the Cav-2 regions pass the threshold value to assign the protein as a disordered protein. 

#### 2.2.3. Hydropathy Analysis

The predicted hydropathic region is defined to be the most likely region associated with the membrane and was calculated by the MPEx Hydropathy Analysis server (Figure 2b). The hydropathic region was formed by residues 70 to 154. The CARC (81–88) domain is present in this region (Figure 1c and Figure 2b). These results support that this section might be inserted or associated with the membrane and might be able to interact with cholesterol. In this region, the server defined three areas as hydropathy predicted segments by the server: 70–88, 98–133, and 139–154 (Figure 2a, blue line).

### 2.3. Far-UV-Circular Dichroism (CD) Spectra of Cav-2

Far-UV-CD spectra of Cav-2 were obtained from 200 nm to 260 nm (Figure 2c). Two negative bands at 208 and 222 nm wavelengths, characteristic of a well-folded protein with a mainly alpha-helix secondary structure, were observed in these spectra. Analysis of the secondary structure with BestSel, as indicated in the Material and Methods, show the following types and percentages of secondary structure: 55.1% of regular helix, 21.1% of distorted helix, 0.2% of right twist, 7.7% of turn and 15.8% of another type of secondary structure. These data support that the isolated protein presents some degree of folding. Moreover, the effect of phospholipids on the Cav-2 secondary structure was also measured. We monitored the CD ellipticity changes by measuring the changes on the [ϴ]_222nm_, which is assigned to weak broad n → π* transitions associated with peptide bonding. Far-UV-CD spectra of the protein, in the presence of PC SUVs at 320 µM (dashed line) and 640 µM (dotted line) and PC SUVs (320 µM) supplemented with cholesterol 5% (+symbols) and in the absence of lipids (continuous line), is shown in Figure 2. A decrease in the protein ellipticity was found by the presence of lipids concerning the control (Figure 2), which also correlates with a possible protein conformational change between two conformation shapes previously described for Cav-1 [43]. In the absence of lipids, Cav-2 presents a ‘w’-shaped with troughs around 222 nm and 208 nm, which indicates the presence of α-helical structures. This protein shape was kept in the presence of SUVs 320 µM and SUVs 320 µM supplemented with cholesterol 5% but tended to disappear in the presence of SUVs 640 µM towards a beta barrel-like conformation characterized by a ‘v’-shaped spectra with a trough around 217–220 nm [43,44]. A [ϴ]_208nm_ > [ϴ]_222nm_ has been attributed to the presence of α-helical segments on the protein [45], a condition that seems to fit with this scenario, as it accounts for the case of Cav-2 and the protein incubated with SUVs 320 µM and SUVs at the same concentration supplemented with cholesterol. The ratio of the two minima ([ϴ]_222nm_/[ϴ]_208nm_) provides an estimate of the extent of coiled-coil formation. A ratio >1 indicates well-defined coiled coils, whereas lower values indicate a less defined coiled-coil structure. The calculation of this index for Cav-2 is indicative of the presence of isolated helix segments ([ϴ]_222nm_/[ϴ]_208nm_ = 0.96) [45]. In the presence of SUVs (320 µM), we observed a change in the helical content towards a coil/coil conformation ([ϴ]_222nm_/[ϴ]_208nm_ = 1.12), and this effect was even more pronounced in the presence SUVs at a lipid concentration (640 µM) 20 times higher than the amount of used protein (32 µM) ([ϴ]_222nm_/[ϴ]_208nm_ = 1.22). The presence of cholesterol (5%) in SUVs (320 µM) induced a reversion of the effect found in experiments performed only with phospholipid (320 µM) [ϴ]_222nm_/[ϴ]_208nm_ = 0.83, supporting the formation of isolated alpha helixes. 

### 2.4. Lipids Stabilize the Strucuture Cav-2 

**Figure 3:**
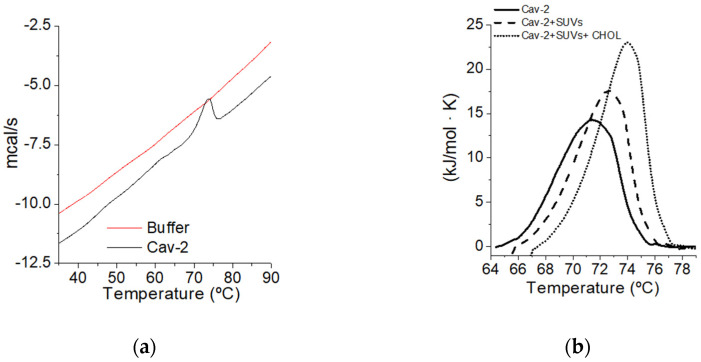
Cav-2 (120 μM) was prepared and run against buffer: sodium phosphate buffer 25 mM pH 7. to adjust optional conditions above the noise (panel **a**). After buffer subtraction, a comparison of the signal between this signal (continuous line) and that obtained in the presence of SUVs (1.2 mM) (dashed line) and SUVs (1.2 mM) in the presence of cholesterol (5%) (dotted line) was performed (panel **b**). Differential scanning calorimetry measurements were carried out using a Nano DSC TA differential scanning calorimeter, operated with 700 µL of the sample at a scanning rate of 0.5 °C/min. The DSC spectra shown in this figure are representative of triplicate experiments.

Differential scanning calorimetry experiments were used to analyze the protein stability of Cav-2 in the presence of lipids (Figure 3). First, the signal was calibrated to obtain a compatible signal-to-noise ratio (panel a). A differential thermogram of Cav-2 in the buffer is shown in panel a. The thermograms of Cav-2 obtained in the absence (continuous black line) and those obtained in the presence of SUVs (1.2 mM) (dashed line) and SUVs (1.2 mM) plus 5% cholesterol (grey line) are shown in panel b. These results indicate that in the presence of lipids, the critical temperature and enthalpy of Cav-2 unfolding increase and that these are effects enhanced by the cholesterol supplementation of SUV. Therefore, these results point out that SUVs stabilize Cav-2 against protein unfolding, and this effect is potentiated by supplementing SUVs with 5% cholesterol. 

### 2.5. Cav-2 Interaction with Phospholipid Bilayers and Effect of Cholesterol

**Figure 4:**
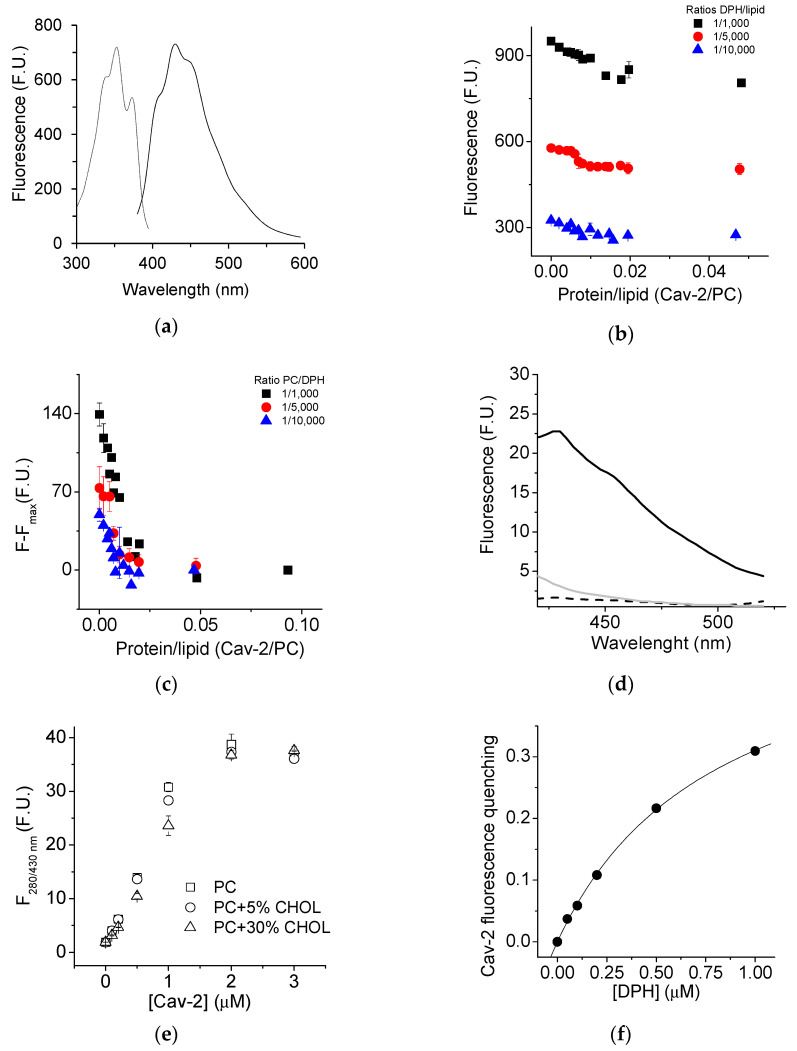
Measurement of Cav-2 interaction with membranes by FRET. Emission (solid line) and excitation (dotted line) spectra were obtained with fixed emission and excitation wavelengths at 452 nm and 354 nm, from SUVs made with egg yolk lecithin 50 μM of egg yolk lecithin and 0.05 μM of DPH concentration (panel **a**). Fluorescence quenching of DPH dependence upon Cav-2 concentration measured with a constant lipid concentration is shown in (panel **b**). DPH was incorporated into PC SUVs at ratios shown in the figure legend keeping constant the PC SUVs concentration at 50 μM for each experiment. Cav-2 was added to the assay and the ratio Cav-2/lipid was calculated for each point to plot these data in the figure. Data normalization of previous figure by the maximum quenching is shown in (panel **c**) by subtracting the maximum quenching exerted by Cav-2 from the initial fluorescence F0 (panel **c**). Emission spectra of Cav-2 (3 μM) (grey line), LUVs (50 μM) supplemented with DPH (0.05 μM) (dashed line), and Cav-2 (3 μM) mixed LUVs (50 μM) supplemented with DPH (0.05 μM) (continuous line) were measured with a fixed excitation wavelength of 280 nm with excitation and emission slits of 10 nm each (panel **d**). The dependence upon caveolin concentration of the fluorescence recorded with fixed excitation and emission wavelengths of 280 and 430 nm from LUVs (50 μM) supplemented with different cholesterol concentrations (0%, 5%, and 30%, shown by open squares, open circles, and open triangles, respectively) and 0.05 μM of DPH is shown in (panel **e**). Calculation of the FRET efficiency by measuring at fixed excitation and emission wavelength of 280 nm and 340 nm, respectively, the DPH fluorescence dependence upon caveolin concentration in LUVs (50 μM) supplemented with DPH (0.05 μM) measured (panel **f**).

1,6-diphenyl-1,3,5-hexatriene (DPH) was used to gain further insight into the interaction between Cav-2 and SUVs. The excitation and emission spectra of DPH (0.05 µM) incorporated into the SUVs (50 µM) is shown in Figure 4a. Cav-2 induced a decrease in the DPH fluorescence incorporated into SUVs at different lipid: dye ratios (1000:1, 5000:1, 10000/1 showed by black, red, and blue symbols, respectively) that were dependent upon the protein concentration (Figure 4b,c). Based on the signal/noise ratio, an ideal 1000:1 lipid: dye ratio was selected for the subsequent experiments (Figure 4b,c).

Since SUVs have a large membrane curvature [46], we used LUVs to study the interaction between Cav-2 and lipid bilayers, to exclude the possibility that this could be critical for Cav-2 interaction with lipid bi-layers. We prepared LUVs (50µM lipid concentration) loaded with DPH (1000:1 lipid: dye ratio) supplemented with cholesterol at different molar fractions (0%, 5%, and 30%). The emission spectra of Cav-2 (3 µM) (grey line), LUVs loaded with DPH (ratio DPH/lipid 1/1000) (dashed line), and Cav-2 plus LUVs loaded with DPH at the same concentrations (black line) have been obtained with a fixed excitation wavelength of 280 nm (Figure 4d). The emission spectra of Cav-2 (grey line) and LUVs supplemented with DPH (dashed line) showed that these samples present a very weak fluorescence emission at 452 nm, the emission wavelength of DPH. In contrast, the fluorescence emission of Cav-2 samples plus LUVs loaded with DPH (continuous line) revealed a large fluorescence increase in the 420–500 nm wavelength range, as shown in Figure 4d. This fluorescence increase pointed out the existence of FRET between the Cav-2 tryptophan residues (donor) and the molecules of DPH (acceptor) incorporated into the lipid bilayer. Thus, we measured the dependence of DPH fluorescence incorporated into LUVs, supplemented with different molar ratios of cholesterol, upon Cav-2 concentration (Figure 4e) using an excitation wavelength of 280 nm. Noteworthy, we did not find significant differences in these measurements using LUVs supplemented with 0%, 5%, and 30% cholesterol/lipid molar ratios. Therefore, the formation of Cav-2:LUVs complexes does not seem to depend upon the cholesterol content of the lecithin lipid bilayer. These results also show that 0.75 ± 0.1 μM Cav-2 produces half of the maximum increase in FRET-mediated DPH fluorescence in LUVs with 50 μM total lipids in the cuvette.

**Figure 5:**
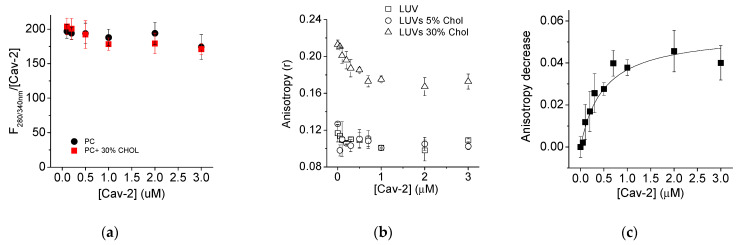
Steady-state anisotropy experiments. (Panel **a**): Dependence of the anisotropy of Cav-2 tryptophan fluorescence (excitation and emission wavelength of 280 nm and 340 nm, respectively) upon the concentration of Cav-2 in the presence of PC-LUVs and PC-LUVs supplemented with 30% of cholesterol. (Panel **b**): dependence upon Cav-2 concentration of the steady-state anisotropy of DPH incorporated into LUVs supplemented with different cholesterol concentrations: 0% (open squares), 5% (open circles), and 30% (open triangles). The measurements of DPH fluorescence anisotropy were performed with excitation and emission wavelengths of 354 and 452 nm, respectively, in sodium phosphate buffer 25 mM (pH 7.0) in a 2 mL quartz cuvette of 1 cm path length and using a Perkin–Elmer 650-40 with polarizers in parallel and perpendicular positions. (Panel **c**): Decrease in DPH anisotropy induced by Cav-2 in LUVs with 30% cholesterol and data fitting to a hyperbolic equation y = P1. x/(Kd + x), where P1 is the maximum binding of caveolin at saturation, × is the concentration of Cav-2, and Kd the dissociation constant. This figure shows the average of experiments performed by triplicate ± S.D.

The FRET efficiency at saturation by Cav-2 of the DPH fluorescence with excitation at 280 nm of LUVs loaded with DPH (ratio DPH/lipid 1/1000) can be calculated from the quenching of the donor fluorescence, Cav-2 intrinsic fluorescence (Figure 5a). Our results yield a FRET efficiency of 16% at a saturating concentration of Cav-2 with LUVs loaded with DPH at a ratio of DPH/lipid 1/1000. However, our measurements did not allow us to calculate the distance between the FRET pair finely. To further refine and show the proximity between Cav-2 tryptophan residues and DPH, we measured the dependence upon the DPH concentration incorporated into LUVs of the Cav-2 tryptophan fluorescence quenching Figure 4f). These results fit well (R^2^ = 0.99) to a hyperbolic equation, as indicated in the legend of this Figure, and as expected for the dependence of the efficiency of FRET upon the density of acceptors in a lipid bilayer [47]. At a concentration of 2 μM DPH, i.e., at a 1:1 molar ratio of Cav-2:DPH, the fit gives an extent of quenching of the donor fluorescence by FRET close to 60%. Our data support the extensive adsorption of Cav-2 onto LUVs.

We experimentally assessed the penetration of Cav-2 into the core of the lipid bilayer by analysis of the fluorescence anisotropy of DPH incorporated into LUVs with different molar ratios of cholesterol: lecithin and its dependence upon interaction with Cav-2. As indicated in the Materials and Methods, the steady-state fluorescence anisotropy (*r*) has been calculated from fluorescence measurements using polarizers. The results of the steady-state anisotropy measurements after the addition of different concentrations of Cav-2 to LUVs with molar ratios of cholesterol:lecithin 0, 5, and 30% are shown in Figure 5b. In the absence of Cav-2, the *r*-value of DPH incorporated into LUVs was close to 0.12 for LUVs and LUVs supplemented with 5% of cholesterol, and this value increased in LUVs supplemented with 30% of cholesterol up to 0.21. The addition of increasing concentrations of Cav-2 did not induce changes in the *r*-value of LUVs samples (open squares) and LUVs samples supplemented with cholesterol 5% (open circles). In samples with 30% of cholesterol, Cav-2 induced a concentration-dependent decrease in the *r*-value from 0.21 to 0.17. The results fit well (R^2^ = 0.96) with a hyperbolic equation of the decrease in the anisotropy induced by the presence of Cav-2 was used to calculate and shows that a concentration of 0.48 ± 0.14 μM Cav-2 produced half of the maximum change of anisotropy (Figure 5c). This value is close to the Cav-2 concentration, which produces half of the maximum increase in FRET-mediated DPH fluorescence in LUVs containing 30% cholesterol (Figure 4e). 

## 3. Discussion

In this paper, we expressed and purified recombinant human Cav-2 in *E. coli* with a yield of 4.2 mg/L in culture media. Cav-2 is proposed as an adequate protein model to study caveolae and the interaction of Cav isoforms with protein and lipids at these domains. A significant disadvantage of using Cav-1 for interaction studies is its high tendency to form high molecular weight oligomers [38,38,39]. In contrast, Cav-2 has a lower tendency than Cav-1 to aggregate in high molecular weight oligomers [9,37]. 

Some of the Cav-2 structural features can be suggested in the in silico homology analysis of the different Cav isoforms, and some of the functionalities observed for Cav-1 were expected for Cav-2. Previous reports indicated that there is a relatively high degree of identity between Cav-1 and -3, whereas Cav-2 is by far the most divergent of the three proteins [14], a relevant homology degree is found between Cav-1 and -2 as observed by our sequence alignment (Supplementary Figure S2). The interaction between Cav-2 and membranes and cholesterol is expected since the protein presents a hydropathic region in the same area where the CRAC domain of Cav-1 is localized (Figure 1c). NMR studies of the shorter CARC domain of the Cav-1(V94-R101) peptide and the homologous sequence of Cav-2 (I79-F87) have shown a similar behavior and the dependence of L102 on the amino acid variations occurring in Cav-2 peptide structure and localization in DPC micelles [40]. Although the Cav-2 sequence I79SKYVMYKF87 does not correlate with the predicted sequence associated with the CRAC domain, the addition of the leucine 88 allows us to predict that the responsible sequence for cholesterol binding in this protein is a CARC domain (K/R)-X1−5-(Y/F)-X1−5-(L/V): in this case K81YVMYKFL88 (Supplementary Figure S3), with the opposite orientation sequence of the CRAC domain [16].

We found a high consensus between Cav-1 and Cav-2 in some amino acids that regulate protein function, i.e., the S37 residue of Cav-1, whose phosphorylation has been shown to modulate the interaction with other protein partners in *in vivo* experiments [47] or P110 that is in charge of Cav-1 turning into a “re-entrant” in the membrane, as part of the helix-break-helix structure [48]. As observed, the region between amino acid residues 68 and 130 in the alignment, which corresponds with region 53–115 of Cav-2, is the region with the highest homology between the different isoforms (Supplementary Figure S2). In this area of the sequence, the helix-break-helix structure of Cav-1 has been localized [49], as part of the IMD (102–134) [14] and a certain degree of homology is shown for the Cav-2 segment (86–119). The hydropathy analysis also predicted that the Cav-2 domain located between 70 and 148 residues is the most likely to be associated with the membrane, where the CARC (81–88) domain is located (Figure 1c and Figure 2b). These results support the potential role of this Cav-2 region interacting with the membrane and cholesterol. It is important to notice that the interacting domain with cholesterol is inside the binding motif (CBM) (Figure 1c), and this suggests that accessibility may be dependent upon the presence of ligands that can also associate with this domain [10]. 

The far UV-CD spectra support that the protein is folded in the absence of lipids with 55.1% as a regular helix, 21.1% as a distorted helix, 0.2% as a right twist, 7.7% as a turn and 15.8% of another type of secondary structure, as predicted by the simulation of the far-UV CD spectra with Bestsel. This correlates with the 46% degree of disorder predicted with ANCHOR and IUPRED server by analysis of the amino acid sequence of Cav-2 located at the amino-terminal end and delimited by residues 0 to 75. The prediction of β-sheet structures agrees with their extent in Cav-1 within the CDS (between positions 87 and 96) and a computational study that predicted an antiparallel β-hairpin motif in Cav-1 residues 84–94 [50,51]. It was also shown for the case of Cav-1 that the peptide 82–109 would contain a mixture of β-sheets and α-helical structures [51]. In the same study, P110 of Cav-1 was proposed to be the responsible residue for the turn in the “re-entrant” helix, whose conformation is stabilized by the interaction with residues in the 103–122 segment [48] part of the helix-break-helix structure [49]. This area presents homology with the corresponding sequence in Cav-2, showing a conserved proline residue at that location (Supplementary Figure S2).

Cav-2 suffers a conformational change in the presence of lipids towards a coil-coiled conformation dependent on the lipid concentration. In the presence of cholesterol, partial reversion of this conformation was found based on the obtained [ϴ]_222nm_/[ϴ]_208nm_ ratios that support the formation of a largely alpha helix conformation. Based on these results and those predicting the shift from a coil-coil conformation towards alpha helixes in Cav-2 upon cholesterol binding, we can compare these data to those reported for Cav-1. Two portions of the TM domain (96–136) have been described to form an alpha helix-break-alpha helix secondary structure with the helices at Cav-1 formed by residues 97–107 and 111–129 (Supplementary Figure S3). These segments correspond to the Cav-2 residues 82–92 and 96–115. Based on CD data, the Cav-1 TM domain maintains 57% of the α-helical secondary structure after reconstitution in LMPG micelles. Taking into account the high consensus between Cav-1 (111–129) and Cav-2 (96–115) (Supplementary Figure S3, amino acid residues labeled in red in the alignment) and the increased alpha helix content based on our far-UV-CD spectra (based on [ϴ]_222nm_/[ϴ]_208nm_), we can predict that the Cav-2 segment 96–115 probably present an alpha helix secondary structure after protein reconstitution in lipids.

Moreover, a comparison of the DSC data obtained with Cav-2 in different conditions (Figure 3) shows a higher critical unfolding temperature (Tm) and unfolding enthalpy in the presence of lipids. Therefore, the results support that the protein acquires a more stable conformation in the presence of lipids with a huge effect in terms of stability, something expected for reconstituted membrane proteins concerning their state upon solubilization in aqueous solutions. Moreover, the DSC results show that the supplementation of SUVs with 5% cholesterol further potentiates the increase in Tm and enthalpy of the unfolding process.

The results of fluorescence experiments using SUVs and LUVs revealed that Cav-2 extensively binds to lecithin lipid bilayers. Moreover, the fact that there is significant FRET between the tryptophan residues of Cav-2 and DPH inserted in the lipid bilayer points out that Cav-2 domains containing these amino acids are directly involved in the interaction between Cav-2 and lecithin lipid bilayers. Cav-2 tryptophan residues (W) are located at positions 70, 113, and 133 in the protein sequence (Supplementary Figure S1), and the FRET experiments between Cav-2 tryptophan residues and DPH are consistent with the adsorption of this protein domain at the lipid–water interface. Thus, FRET data strongly support an interaction between the C-termini of Cav-2 with the membrane, as shown with Cav-1 [52]. We did not find any difference in this interaction when these measurements were performed in the absence of cholesterol (Figure 4e), which supports that cholesterol does not interfere with the protein adsorption to the lipid–water interface of the lecithin lipid bilayer. However, steady-state anisotropy measurements of DPH detected that Cav-2 interacted with LUVs with 30% cholesterol differently than with LUVs formed with egg yolk lecithin or egg yolk lecithin with 5% cholesterol. Cholesterol increased the steady-state anisotropy of DPH incorporated in egg yolk lecithin LUVs containing 30% cholesterol with respect to the steady-state anisotropy experiments of egg yolk lecithin LUVs or only 5% cholesterol. This behavior has been attributed to a reduced rotational diffusion of the rigid molecular probe that correlates with decreased bilayer fluidity [53]. The addition of Cav-2 to LUVs containing 30% cholesterol decreased the DPH anisotropy, indicating that Cav-2 makes the inner core of these lipid bilayers where DPH is located more fluid or disordered [54,55]. The addition of Cav-2 to LUVs containing 30% cholesterol decreased the DPH anisotropy, indicating that Cav-2 is incorporated into cholesterol-rich domains and makes them more fluid or disordered. Thus, Cav-2 is likely to interact with cholesterol, as shown earlier for Cav-1 in biological membranes [15]. Cav-2 induced decreases in the steady state anisotropy of lecithin LUVs containing 30% cholesterol only to values that are still clearly higher than those measured for lecithin LUVs or lecithin LUVs containing 5% cholesterol. The Cav-2-induced decrease in DPH anisotropy in LUVs with 30% cholesterol is about half of the increase in anisotropy produced by 30% cholesterol in lecithin LUVs relative to lecithin LUVs without cholesterol. As Cav-2 is added to preformed LUVs, this result is consistent with the interaction of Cav-2 only with half of the cholesterol-rich domains in the lipid bilayer, i.e., those available from the external leaflet of the LUVs lipid bilayer. 

## 4. Materials and Methods

### 4.1. Cav-2 Cloning 

A commercially available construct for CAV2 (Sino Biological Inc. cat#HG14521-G; Eschborn, Germany;) with the inserted sequence for the alpha isoform of human Cav-2 cDNA was used as a template for PCR; the product was inserted into the pET-22b vector. The primers used in the PCR were selected to incorporate a thrombin cutting site to release the His-tag when needed in our experiments. Forwards Primers (0.1 μM) (FW-5′-CAATGCCATGGGGCTGGAGACG-3′ and RW-5′CCCAAGCTTGCCCCGTCCATCCTGGCTCAGTTGCAG-3′) and the commercial plasmid (20 ng) were in the presence of an enzyme (NZY proof DNA polymerase kit cat#MB14601, NZYtech; Lisboa, Portugal) and buffer (MgCl2 1.5 mM, dNTPs (0.25 mM) and enzyme (NZY proof DNA polymerase kit cat#MB14601, NZYtech). The adjusted set for PCRs was: 30 s at 95 °C, 30 s at 58 °C, and 60 s at 72 °C for denaturation, annealing, and extension steps. The insert was designed to add the cutting sites for the NcoI and HindIII restriction enzymes to be inserted into the multi-cloning site. Preparation and purification of the linearized plasmid and PCR product were performed as indicated by the commercial suppliers of the restriction enzymes (Thermo Fisher Scientific; Carlsbad, CA, USA). pET-22b vector dephosphorylation was performed with TSAP thermosensitive alkaline phosphatase (cat#M9910, Promega Biotech Ibérica SL; Madrid, Spain). Insert ligation into the plasmid was performed with a Rapid DNA Ligation kit (cat#11635379001, Roche; Mannheim, Germany). Transformation into *E. coli* DH5α strains were accomplished, and positive colonies were picked and grown in liquid LB medium with Ampicillin (0.1 mg/mL). Plasmid purification was achieved to obtain a stock solution of the cloned plasmid for CAV2 (hCAV2) and stored at −80 °C.

### 4.2. Cav-2 Expression in E. coli 

One microliter of CAV2pET22b plasmid was added to 50μL of competent Rosetta–Gammi 2 cells, and the mix was incubated for 30 min in ice. Cells were incubated in a 42 °C bath for 45 s (thermic shock) and incubated on ice for another 5 min. Finally, 200 μL of SOC medium was added, and the cells were grown for one hour at 37 °C in a continuous rotatory shaker at 190 rpm. Transformed cells were grown in a solid LB medium supplemented with ampicillin (0.1 mg/mL) and chloramphenicol 0.035 mg/mL).

A single colony was picked up and transferred into 5 mL of LB supplemented with ampicillin and chloramphenicol and grown overnight. The media was added into 100 mL of fresh LB media containing antibiotics and kept at 37 °C with continuous rotatory shaking at 190 rpm until 0.6 D.O. absorbance was reached. Isopropyl β-D-1-thiogalactopyranoside (IPTG) (0.5 mM) was added to the cultures at 0.6 D.O. After induction, cell cultures were left growing at 25 °C overnight. Cultures were centrifuged for 15 min at 6000× *g* at the selected times, and pellets were resuspended with lysis buffer: Tris 50 mM, NaCl 100 mM, PMSF 1 mM, Lysozyme 0.1 mg/mL, DOC 0.25%, DNAse 0.05 mg/mL and MgCl_2_ 50 mM under shaking at 4 °C for four hours. 

### 4.3. Purification

#### 4.3.1. Sephadex G75

Samples were centrifuged at 8000× *g* for 30 min, and the supernatant was taken and loaded onto a Sephadex G75 (2.5 cm d × 50 cm l), previously equilibrated with Tris 150 mM at pH 8. Chromatography was run at a constant flux rate (2 mL/min and eluting fractions were characterized by SDS-PAGE to determine the fractions containing bands corresponding to the molecular weight of Cav-2α. These fractions were combined and dialyzed several times against 5 L of Tris 5 mM at pH 8 and concentrated using a Vivaspin Turbo 15 (3 kDa cut off) (Sartorious; Gloucestershire, UK).

#### 4.3.2. Purification Using the His-Trap Affinity Column and Native Conditions

Concentrated samples were supplemented with Tx-100 2%, CTAB 0.4 mM, and imidazole 7 mM at pH 8.0 and were loaded onto a packed HisTrap column (2 cm d × 4.5 cm l) (GEHealthcare) previously equilibrated with binding buffer: Tris 5 mM, at pH 8.0. The sample was incubated overnight, and the column was washed with Tris 20 mM at pH 7.5. Samples containing purified Cav-2α were eluted with eluting Buffer: Tris 20 mM, imidazole 250 mM, CTAB 0.2 mM, and TX-100 0.4 mM at pH 7.5. All the steps were performed at a flux rate of 2 mL/min using the AKTA CV-950 (UV-900, P-900) Amersham Pharmacia biotech (041854) and fraction collector (Pharmacia LKB-Redifrac 2928, GE Healthcare Bio-Sciences AB; Uppsala, Sweden). After selecting samples containing purified Cav-2, fractions were mixed and extensively dialyzed against Tris 5 mM, pH 8, and concentrated using a Vivaspin Turbo 15 filter (cut-off 5 kDa). Finally, 30% glycerol was added to the purified protein, and samples were stored and frozen at −80 °C until use.

### 4.4. Preparation of SUVs and LUVs

SUVs were prepared as described in [56]. Chloroform/methanol solutions of egg yolk lecithin and cholesterol were added onto a tube and dried by flux with argon. Once lipids were dried entirely, phosphate buffer 20 mM, pH 7.0 was added, and the solution vortexed. Lipids were exposed to sonication with a titanium-microtip-equipped Hielscher UP200S sonicator for 15 cycles of 10 s sonication, followed to rest. SUVs were stored at room temperature and used the same day they were prepared. 

For LUVs preparation, the Avanti mini extruder was used, following the manufacturer’s instructions at room temperature.

### 4.5. Western Blotting

Western Blot was performed following descriptions indicated anywhere. SDS-PAGE electrophoresis was performed after loading 5.2 μg of purified Cav-2 protein (0.203 mg/mL). The polyacrylamide gel, a 0.2 nm porosity polyvinylidene fluoride (PDVF) membrane, and eight filter sheets are cut with the same gel size.

The PVDF and the gel were incubated in methanol and Transfer Buffer (25 mM Tris, 195 mM Glycine, 20% methanol, pH 8.3) for 5 and 10 min, respectively. The filter sheets and the electro-transfer sponge were wet in the transfer buffer, and the PVDF was in methanol before the transfer sandwich was prepared: sponge, four filter sheets, Gel PVDF, four filter sheets, and sponge. The transfer sandwich was located in the transfer apparatus, and electrophoresis was performed horizontally in the transfer buffer under a 54-mA fixed amperage, at 4 °C, for 18 h 30′.

For blocking and incubation with antibodies, the first membrane was incubated in methanol and Tween Tris-buffered saline (TBST, 50 mM Tris, 150 mM NaCl, 0.2% Tween 20, pH 7.6) for 5 min each. The membrane was blocked for 1 h in 3 mg/mL BSA containing TBST and washed with TBST (5′) and in TBS (no detergent containing TBST) (15′) three times each.

The membrane was incubated for one hour in goat anti-Cav-2 (Santa Cruz, CA, #sc-1858) primary antibody, diluted 1/200 in TBST. The antibody was supplied by Santa Cruz Biotechnology (Santa Cruz, CA, USA). After washing with TBST, the membrane was incubated for another hour in anti-goat IgG-Cy3 (ct. number C2821) secondary antibody diluted 1/5000 in TBST and supplied by Sigma-Aldrich (St. Louis, MO, USA). All the antibodies were used in the dilution range recommended in their technical sheets. The membrane was finally washed and revealed with luminol, as shown in the commercial data sheet.

### 4.6. Circular Dichroism (CD) 

Far-UV Circular dichroism (Far-UV CD) spectra of Cav-2 (64 μM) prepared in phosphate buffer 20 mM pH 7.0 were measured using a Chirascan CD spectrometer (Applied Photophysics circular dichroism spectrophotometer). The experiments were carried out at 25 °C, room temperature, at a scanning rate of 3 sec/nm, and under a nitrogen pressure of 5 L/min during the scan. Spectra were obtained from 260 to 190 nm using three accumulations. A 0.1 mm path length cuvette (High Precision Quartz Suprasil Cell, Hellma Analytics; Müllheim, Germany) and 100 μL of samples were used. In addition, the effect of egg yolk lecithin (PC) formed SUVs supplemented or not with cholesterol 5% were measured with human recombinant Cav-2 (32 μM). A background spectrum containing no buffer or SUVs supplemented or not with cholesterol was subtracted from each corresponding protein spectra. Far UV-CD spectra were analyzed using the BestSel server (http://bestsel.elte.hu/index.php) accessed on 19 November 2022.

### 4.7. Differential Scanning Calorimetry (DSC)

Cav-2 120 µM reconstituted in SUVs prepared with PC (1.2 mM) were prepared in phosphate 20 mM pH7 buffer in the absence and presence of cholesterol (5%). DSC measurements were carried out using a Nano DSC TA differential scanning calorimeter operated with 700 μL of the sample at a scanning rate of 0.5 °C/min. The concentration of protein was always 2 mg/mL. The buffer used in DSC experiments was 20 mM potassium phosphate (pH 7.0). The calorimetry data were analyzed using the NanoAnalyze Data Analysis software (TA instruments; Waters, New Castle, DE, USA).

### 4.8. Fluorescence Intensity and Anisotropy Measurements 

Dimethylformamide solutions of 1,6-diphenyl-1,3,5-hexatriene (DPH) were mixed with chloroform solutions of egg yolk lecithin, and SUVs were prepared as indicated above. For DPH fluorescence measurements using LUVs, DPH was added to already prepared LUVs and allowed for equilibration into the phospholipid bilayer under mild stirring in the fluorescence cuvette for 5 min before starting fluorescence readings. Titration of DPH fluorescence with Cav-2 was performed by adding different concentrations of Cav-2, and fluorescence measurements were taken 2 min after adding Cav-2. Fluorescence measurements and fluorescence spectra were acquired using quartz cuvettes with a 10 nm path length at room temperature with a Perkin–Elmer 650-40 spectrofluorometer. DPH fluorescence measurements were acquired with excitation, and emission wavelengths of 350 and 452 nm, respectively, with excitation and emission slits of 10/10 nm. For Cav-2 intrinsic fluorescence measurements, excitation and emission wavelengths were fixed at 280 and 340 nm, respectively, with excitation and emission slits of 10/10 nm. Fluorescence spectra were recorded at a 60 nm/min scan rate, and three scans were recorded per measurement. 

Steady-state fluorescence anisotropy (r) has been calculated using the following equation [53]: *r* = (I_//_ − G.I_⊥_)/(I_//_ + 2G.I_⊥_), where I_//_ and I_┴_ are the fluorescence intensity with excitation and emission polarizers oriented at 0°/0° and 0°/90°, and G is the grating factor (G = I_90/0_/I_90/90_).

### 4.9. Prediction of Structural Information Obtained by Informatic Tools

Jalview 2.11 was used to visualize the Cav-2 sequence [57]. The Mutalin interface alignment server (http://multalin.toulouse.inra.fr/multalin/) accessed on 9 June 2019, was used to perform the homology analysis of the Cav sequences [58]. The prediction of structurally intrinsically disordered segments was performed with the IUPred2 and ANCHOR2 web-based implementation predictors [41]. The hydropathy analysis was performed by sending the Cav-2 sequence to the Membrane Protein Explorer (MPEx) server (https://blanco.biomol.uci.edu/mpex/), accessed on 9 June 2019 [59], with the selected interfacial Wimley–White scale, a residues window of 19, and bilayer partitioning to water and a bilayer surface potential of 0 mV.

### 4.10. Data Analysis

The data reported in this manuscript are the average of experiments performed by triplicate ± standard deviation (S.D.)

## 5. Conclusions

**Figure 6:**
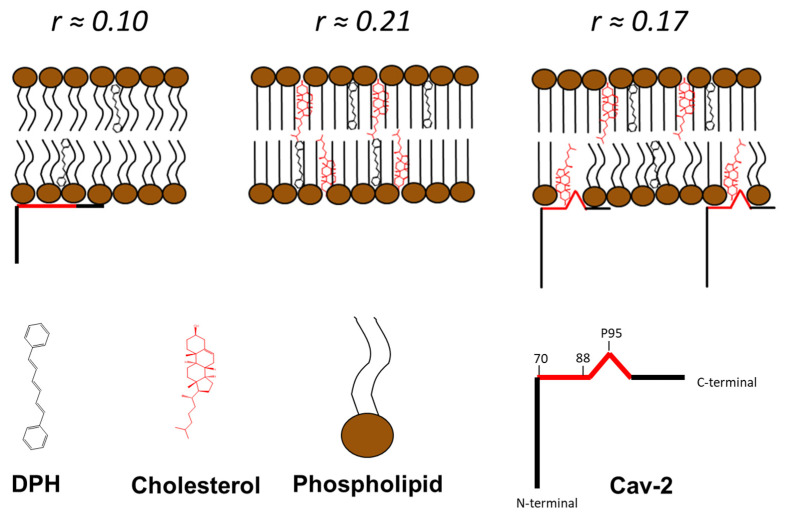
The scheme of the cholesterol redistribution in the membrane induced by Cav-2 is based on steady-state anisotropy experiments.

In summary, the results of bioinformatic prediction models, circular dichroism, differential scanning calorimetry, and fluorescence techniques, demonstrate the interaction of Cav-2 with the lipid bilayer of lecithin liposomes. Circular dichroism and differential scanning calorimetry studies show that this interaction elicits significant structural changes in Cav-2. In addition, our data support that Cav-2 binds and alters cholesterol-rich domains in the membranes (Figure 6), likely through a CARC domain. Therefore, purified recombinant Cav-2 is an alternate new tool to study protein–lipid interactions within caveolae.

## Figures and Tables

**Table 1:** Purification table.

STEP	Protein Amount	Yield (%)
Whole lysate Periplasmic fraction Sephadex G75 Niquel Sepharose	530.1	100
	182.0	34.3
	147.0	27.7
	4.2	1.6

Protein quantified by the Bradford Method. Data in this table represents the amount of protein recovered in each step, using 1 L of the initial overgrowth bacterial culture.

## Data Availability

Data is contained within the article or supplementary material. The raw data presented in this study are available on request from the corresponding author (A.K.S.A.).

## Supplementary Materials

The following supporting information can be downloaded at: https://www.mdpi.com/article/10.3390/ijms232315203/s1.

## References

[1] Palade G.E. (1953). Fine Structure of Blood Capillaries. J. Appl. Phys..

[2] Stillwell W., Stillwell W. (2013). Chapter 11-Long-Range Membrane Properties. An Introduction to Biological Membranes.

[3] Parton R.G., Hanzal-Bayer M., Hancock J.F. (2006). Biogenesis of Caveolae: A Structural Model for Caveolin-Induced Domain Formation. J. Cell Sci..

[4] Pol A., Morales-Paytuví F., Bosch M., Parton R.G. (2020). Non-Caveolar Caveolins-Duties Outside the Caves. J. Cell Sci..

[5] Parton R.G., McMahon K.-A., Wu Y. (2020). Caveolae: Formation, Dynamics, and Function. Curr. Opin. Cell Biol..

[6] Song K.S., Scherer P.E., Tang Z., Okamoto T., Li S., Chafel M., Chu C., Kohtz D.S., Lisanti M.P. (1996). Expression of Caveolin-3 in Skeletal, Cardiac, and Smooth Muscle Cells. Caveolin-3 Is a Component of the Sarcolemma and Co-Fractionates with Dystrophin and Dystrophin-Associated Glycoproteins. J. Biol. Chem..

[7] Sowa G. (2011). Novel Insights into the Role of Caveolin-2 in Cell- and Tissue-Specific Signaling and Function. Biochem. Res. Int..

[8] Williams T.M., Lisanti M.P. (2004). The Caveolin Proteins. Genome Biol..

[9] Scherer P.E., Okamoto T., Chun M., Nishimoto I., Lodish H.F., Lisanti M.P. (1996). Identification, Sequence, and Expression of Caveolin-2 Defines a Caveolin Gene Family. Proc. Natl. Acad. Sci. USA.

[10] Collins B.M., Davis M.J., Hancock J.F., Parton R.G. (2012). Structure-Based Reassessment of the Caveolin Signaling Model: Do Caveolae Regulate Signaling through Caveolin-Protein Interactions?. Dev. Cell.

[11] Byrne D.P., Dart C., Rigden D.J. (2012). Evaluating Caveolin Interactions: Do Proteins Interact with the Caveolin Scaffolding Domain through a Widespread Aromatic Residue-Rich Motif?. PLoS ONE.

[12] Li S., Couet J., Lisanti M.P. (1996). Src Tyrosine Kinases, Galpha Subunits, and H-Ras Share a Common Membrane-Anchored Scaffolding Protein, Caveolin. Caveolin Binding Negatively Regulates the Auto-Activation of Src Tyrosine Kinases. J. Biol. Chem..

[13] Patel H.H., Murray F., Insel P.A. (2008). Caveolae as Organizers of Pharmacologically Relevant Signal Transduction Molecules. Annu. Rev. Pharmacol. Toxicol..

[14] Razani B., Woodman S.E., Lisanti M.P. (2002). Caveolae: From Cell Biology to Animal Physiology. Pharmacol. Rev..

[15] Yang G., Xu H., Li Z., Li F. (2014). Interactions of Caveolin-1 Scaffolding and Intramembrane Regions Containing a CRAC Motif with Cholesterol in Lipid Bilayers. Biochim. Biophys. Acta.

[16] Fantini J., Barrantes F.J. (2013). How Cholesterol Interacts with Membrane Proteins: An Exploration of Cholesterol-Binding Sites Including CRAC, CARC, and Tilted Domains. Front. Physiol..

[17] Baier C.J., Fantini J., Barrantes F.J. (2011). Disclosure of Cholesterol Recognition Motifs in Transmembrane Domains of the Human Nicotinic Acetylcholine Receptor. Sci. Rep..

[18] Kwon H., Jeong K., Hwang E.M., Park J.-Y., Pak Y. (2011). A Novel Domain of Caveolin-2 That Controls Nuclear Targeting: Regulation of Insulin-Specific ERK Activation and Nuclear Translocation by Caveolin-2. J. Cell. Mol. Med..

[19] Breuza L., Corby S., Arsanto J.-P., Delgrossi M.-H., Scheiffele P., Le Bivic A. (2002). The Scaffolding Domain of Caveolin 2 Is Responsible for Its Golgi Localization in Caco-2 Cells. J. Cell Sci..

[20] Fujimoto T., Kogo H., Ishiguro K., Tauchi K., Nomura R. (2001). Caveolin-2 Is Targeted to Lipid Droplets, a New “Membrane Domain” in the Cell. J. Cell Biol..

[21] Kang C., Hernandez V.A., Hu K. (2017). Functional Interaction of the Two-Pore Domain Potassium Channel TASK-1 and Caveolin-3. Biochim. Biophys. Acta Mol. Cell Res..

[22] Sotgia F., Lee J.K., Das K., Bedford M., Petrucci T.C., Macioce P., Sargiacomo M., Bricarelli F.D., Minetti C., Sudol M. (2000). Caveolin-3 Directly Interacts with the C-Terminal Tail of Beta -Dystroglycan. Identification of a Central WW-like Domain within Caveolin Family Members. J. Biol. Chem..

[23] Venema V.J., Ju H., Zou R., Venema R.C. (1997). Interaction of Neuronal Nitric-Oxide Synthase with Caveolin-3 in Skeletal Muscle. Identification of a Novel Caveolin Scaffolding/Inhibitory Domain. J. Biol. Chem..

[24] Volonte D., McTiernan C.F., Drab M., Kasper M., Galbiati F. (2008). Caveolin-1 and Caveolin-3 Form Heterooligomeric Complexes in Atrial Cardiac Myocytes That Are Required for Doxorubicin-Induced Apoptosis. Am. J. Physiol. Heart Circ. Physiol..

[25] de Almeida C.J.G. (2017). Caveolin-1 and Caveolin-2 Can Be Antagonistic Partners in Inflammation and Beyond. Front. Immunol..

[26] Li S., Okamoto T., Chun M., Sargiacomo M., Casanova J.E., Hansen S.H., Nishimoto I., Lisanti M.P. (1995). Evidence for a Regulated Interaction between Heterotrimeric G Proteins and Caveolin. J. Biol. Chem..

[27] Samhan-Arias A.K., Fortalezas S., Cordas C.M., Moura I., Moura J.J.G., Gutierrez-Merino C. (2018). Cytochrome B5 Reductase Is the Component from Neuronal Synaptic Plasma Membrane Vesicles That Generates Superoxide Anion upon Stimulation by Cytochrome c. Redox Biol..

[28] Samhan-Arias A.K., Gutierrez-Merino C. (2014). Purified NADH-Cytochrome B5 Reductase Is a Novel Superoxide Anion Source Inhibited by Apocynin: Sensitivity to Nitric Oxide and Peroxynitrite. Free Radic. Biol. Med..

[29] Samhan-Arias A.K., Marques-da-Silva D., Yanamala N., Gutierrez-Merino C. (2012). Stimulation and Clustering of Cytochrome B5 Reductase in Caveolin-Rich Lipid Microdomains Is an Early Event in Oxidative Stress-Mediated Apoptosis of Cerebellar Granule Neurons. J. Proteom..

[30] Samhan-Arias A.K., Garcia-Bereguiain M.A., Martin-Romero F.J., Gutierrez-Merino C. (2009). Clustering of Plasma Membrane-Bound Cytochrome B5 Reductase within “lipid Raft” Microdomains of the Neuronal Plasma Membrane. Mol. Cell. Neurosci..

[31] Valério G.N., Gutiérrez-Merino C., Nogueira F., Moura I., Moura J.J.G., Samhan-Arias A.K. (2020). Human Erythrocytes Exposure to Juglone Leads to an Increase of Superoxide Anion Production Associated with Cytochrome B5 Reductase Uncoupling. Biochim. Biophys. Acta (BBA)-Bioenerg..

[32] Mora R., Bonilha V.L., Marmorstein A., Scherer P.E., Brown D., Lisanti M.P., Rodriguez-Boulan E. (1999). Caveolin-2 Localizes to the Golgi Complex but Redistributes to Plasma Membrane, Caveolae, and Rafts When Co-Expressed with Caveolin-1. J. Biol. Chem..

[33] Ostermeyer A.G., Paci J.M., Zeng Y., Lublin D.M., Munro S., Brown D.A. (2001). Accumulation of Caveolin in the Endoplasmic Reticulum Redirects the Protein to Lipid Storage Droplets. J. Cell Biol..

[34] Burgermeister E., Tencer L., Liscovitch M. (2003). Peroxisome Proliferator-Activated Receptor-Gamma Upregulates Caveolin-1 and Caveolin-2 Expression in Human Carcinoma Cells. Oncogene.

[35] Kwon H., Jeong K., Pak Y. (2009). Identification of PY19-Caveolin-2 as a Positive Regulator of Insulin-Stimulated Actin Cytoskeleton-Dependent Mitogenesis. J. Cell. Mol. Med..

[36] Li S., Galbiati F., Volonte D., Sargiacomo M., Engelman J.A., Das K., Scherer P.E., Lisanti M.P. (1998). Mutational Analysis of Caveolin-Induced Vesicle Formation. Expression of Caveolin-1 Recruits Caveolin-2 to Caveolae Membranes. FEBS Lett..

[37] Scherer P.E., Lewis R.Y., Volonte D., Engelman J.A., Galbiati F., Couet J., Kohtz D.S., van Donselaar E., Peters P., Lisanti M.P. (1997). Cell-Type and Tissue-Specific Expression of Caveolin-2. Caveolins 1 and 2 Co-Localize and Form a Stable Hetero-Oligomeric Complex in Vivo. J. Biol. Chem..

[38] Song K.S., Tang Z., Li S., Lisanti M.P. (1997). Mutational Analysis of the Properties of Caveolin-1. A Novel Role for the C-Terminal Domain in Mediating Homo-Typic Caveolin-Caveolin Interactions. J. Biol. Chem..

[39] Sargiacomo M., Scherer P.E., Tang Z., Kübler E., Song K.S., Sanders M.C., Lisanti M.P. (1995). Oligomeric Structure of Caveolin: Implications for Caveolae Membrane Organization. Proc. Natl. Acad. Sci. USA.

[40] Le Lan C., Gallay J., Vincent M., Neumann J.M., de Foresta B., Jamin N. (2010). Structural and Dynamic Properties of Juxta-Membrane Segments of Caveolin-1 and Caveolin-2 at the Membrane Interface. Eur. Biophys. J..

[41] Mészáros B., Erdos G., Dosztányi Z. (2018). IUPred2A: Context-Dependent Prediction of Protein Disorder as a Function of Redox State and Protein Binding. Nucleic Acids Res..

[42] Dosztányi Z., Mészáros B., Simon I. (2009). ANCHOR: Web Server for Predicting Protein Binding Regions in Disordered Proteins. Bioinformatics.

[43] Miles A.J., Wallace B.A. (2016). Circular Dichroism Spectroscopy of Membrane Proteins. Chem. Soc. Rev..

[44] Ranjbar B., Gill P. (2009). Circular Dichroism Techniques: Biomolecular and Nanostructural Analyses- a Review. Chem. Biol. Drug Des..

[45] Andersen N.H., Liu Z., Prickett K.S. (1996). Efforts toward Deriving the CD Spectrum of a 3(10) Helix in Aqueous Medium. FEBS Lett..

[46] Parton D.L., Klingelhoefer J.W., Sansom M.S.P. (2011). Aggregation of Model Membrane Proteins, Modulated by Hydrophobic Mismatch, Membrane Curvature, and Protein Class. Biophys. J..

[47] Stryer L. (1978). Fluorescence Energy Transfer as a Spectroscopic Ruler. Annu. Rev. Biochem..

[48] Aoki S., Thomas A., Decaffmeyer M., Brasseur R., Epand R.M. (2010). The Role of Proline in the Membrane Re-Entrant Helix of Caveolin-1. J. Biol. Chem..

[49] Lee J., Glover K.J. (2012). The Transmembrane Domain of Caveolin-1 Exhibits a Helix-Break-Helix Structure. Biochim. Biophys. Acta.

[50] Spisni E., Tomasi V., Cestaro A., Tosatto S.C.E. (2005). Structural Insights into the Function of Human Caveolin 1. Biochem. Biophys. Res. Commun..

[51] Hoop C.L., Sivanandam V.N., Kodali R., Srnec M.N., van der Wel P.C.A. (2012). Structural Characterization of the Caveolin Scaffolding Domain in Association with Cholesterol-Rich Membranes. Biochemistry.

[52] Schlegel A., Lisanti M.P. (2000). A Molecular Dissection of Caveolin-1 Membrane Attachment and Oligomerization. Two Separate Regions of the Caveolin-1 C-Terminal Domain Mediate Membrane Binding and Oligomer/Oligomer Interactions in Vivo. J. Biol. Chem..

[53] Lakowicz J.R. (2010). Principles of Fluorescence Spectroscopy.

[54] de Meyer F., Smit B. (2009). Effect of Cholesterol on the Structure of a Phospholipid Bilayer. Proc. Natl. Acad. Sci. USA.

[55] Aguilar L.F., Pino J.A., Soto-Arriaza M.A., Cuevas F.J., Sánchez S., Sotomayor C.P. (2012). Differential Dynamic and Structural Behavior of Lipid-Cholesterol Domains in Model Membranes. PLoS ONE.

[56] Samhan-Arias A.K., Tyurina Y.Y., Kagan V.E. (2011). Lipid Antioxidants: Free Radical Scavenging versus Regulation of Enzymatic Lipid Peroxidation. J. Clin. Biochem. Nutr..

[57] Waterhouse A.M., Procter J.B., Martin D.M.A., Clamp M., Barton G.J. (2009). Jalview Version 2—A Multiple Sequence Alignment Editor and Analysis Workbench. Bioinformatics.

[58] Corpet F. (1988). Multiple Sequence Alignment with Hierarchical Clustering. Nucleic Acids Res..

[59] Snider C., Jayasinghe S., Hristova K., White S.H. (2009). MPEx: A Tool for Exploring Membrane Proteins. Protein Sci..

