# Supplementary material for: Biochemical and Biophysical Characterization of the Caveolin-2 Interaction with Membranes and Analysis of the Protein Structural Alteration by the Presence of Cholesterol
Source: Int J Mol Sci. 2022 Dec 2;23(23):15203. doi: 10.3390/ijms232315203 (PMC9736327; doi:10.3390/ijms232315203)
Supplement: Supplementary file 1 [file ijms-23-15203-s001.zip › Captions for Supplementary Figures.pdf]

Captions for Supplementary Figures

Supplementary Figure S1. Caveolin-2 alpha isoform linear amino acid sequence .

Supplementary Figure S2. Alignment of sequences of different caveolin isoforms with Mutalin e alignment server. The following sequences were used in this study: Cav-1 human isoforms (α- and β- isoforms; Unitprot #Q031352 and # Q03135-1, respectively); Cav-2 human isoforms (α- and β- isoforms ; Unitprot# P51636-1 and t# P51636-2, respectively) and caveolin C (Unitprot# P51636-3)]. Hight consensus areas are labeled in red (>90% homology) and low consensus areas in blue (50% > homology).

Supplementary Figure S3. Comparation between the location of some of the domains described for Cav-2 and those described here for Cav-2for the aligned sequences. The analyzed areas are 81- 149 for Cav-1 (Q03135) and 66-135 for Cav-2 (P51636).
