# Supplementary figures and images for: Biochemical and Biophysical Characterization of the Caveolin-2 Interaction with Membranes and Analysis of the Protein Structural Alteration by the Presence of Cholesterol
Source: Int J Mol Sci. 2022 Dec 2;23(23):15203. doi: 10.3390/ijms232315203 (PMC9736327; doi:10.3390/ijms232315203)

## Slide 1
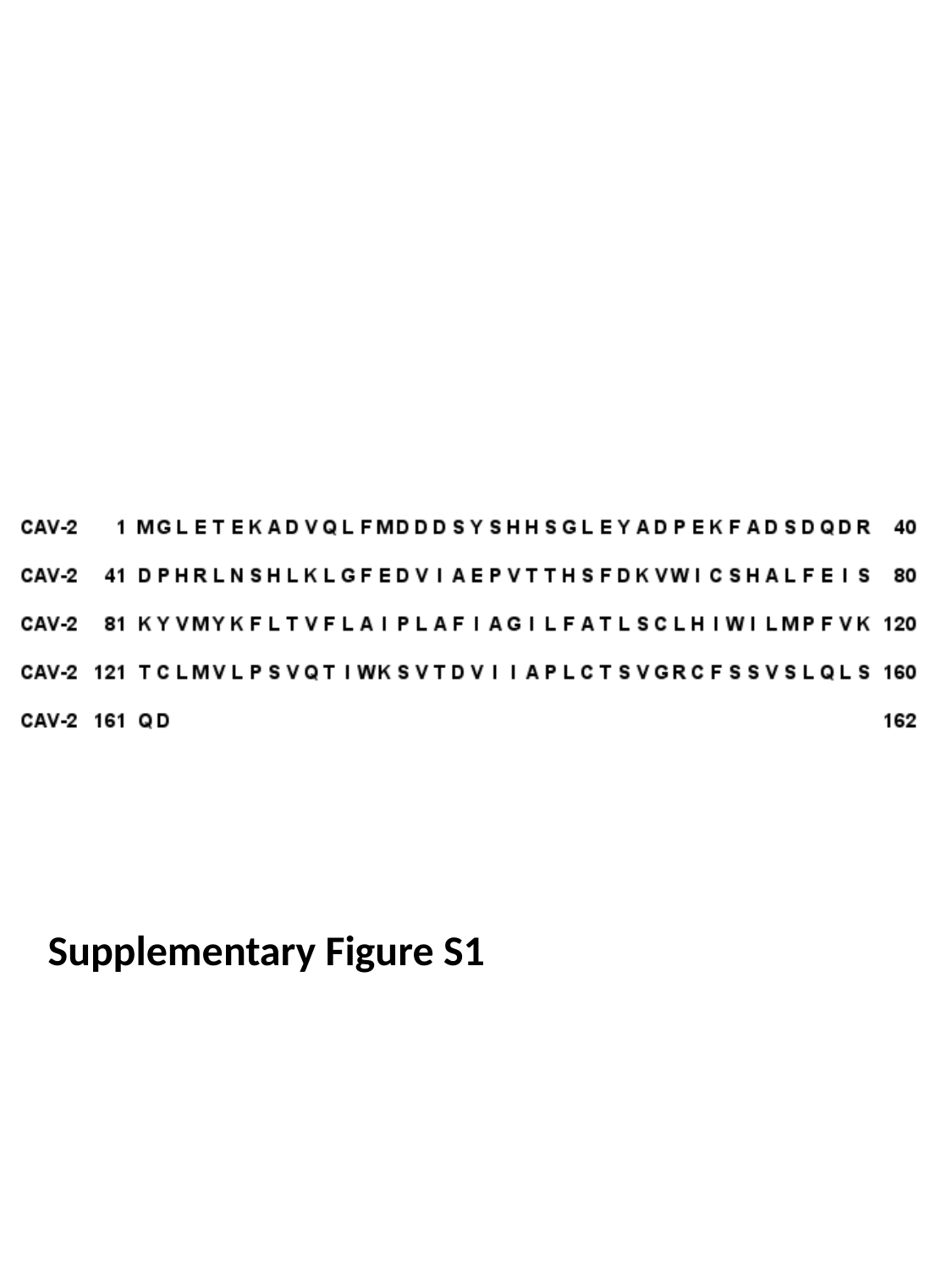

Supplementary Figure S1

## Slide 2
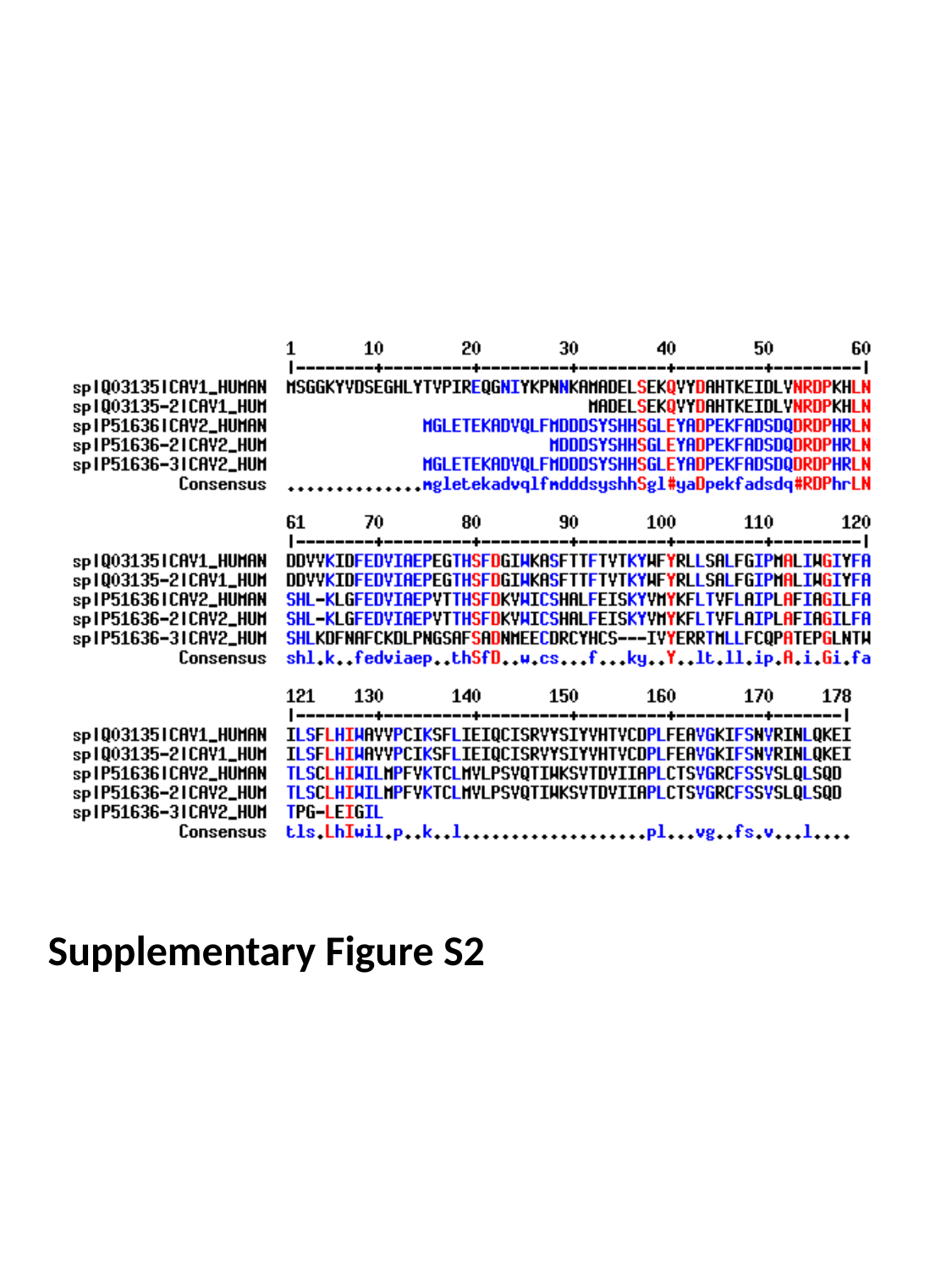

Supplementary Figure S2

## Slide 3
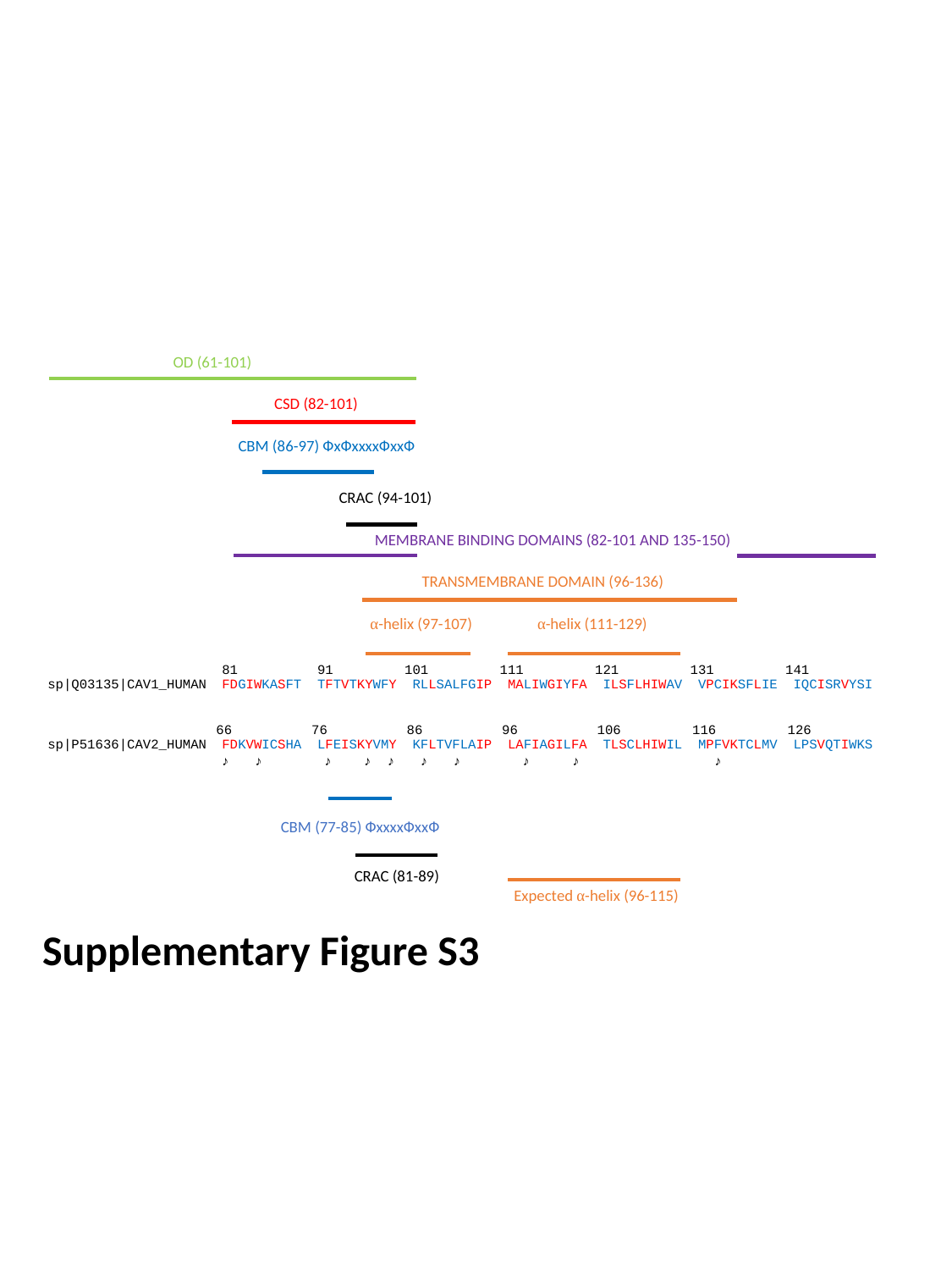

Supplementary Figure S3

Supplement: Supplementary file 1 [file ijms-23-15203-s001.zip › Supplementary Figures.pptx]
